# Assessment of the usefulness of presepsin (soluble CD14 subtype) in septic patients

**DOI:** 10.1186/cc10639

**Published:** 2012-03-20

**Authors:** T Nishida, H Ishikura, A Murai, Y Irie, R Yuge, T Kamitani, S Endo

**Affiliations:** 1 Fukuoka University Hospital, Fukuoka City, Japan; 2 Iwate Medical University, Iwate, Japan

## Introduction

Sepsis is a life-threatening condition that is characterized by a whole-body inflammatory state. The early diagnosis and treatments of sepsis will improve the outcome of the patients. The aims of this study were to investigate the most useful biomarkers which are serum levels of soluble CD14 subtype (sCD14-ST) named presepsin, procalcitonin (PCT), IL-6, and C-reactive protein (CRP) as markers for early diagnosis of sepsis.

## Methods

A single-center, prospective, observational study. Patients who had one or more systemic inflammatory response syndrome (SIRS) criteria were included in this study. The blood samples for measuring the markers were collected and the severity of sepsis was evaluated at the time of admission and every other day for a week. Eighty-four patients were enrolled for this prospective study from June 2010 to June 2011.

## Results

Eighteen were SIRS and 42 were sepsis at the time of registration. In the receiver operating characteristics (ROC) analysis, the area under the curve (AUC) to distinguish sepsis was the highest for presepsin (0.92) followed by IL-6 (0.89), PCT (0.88), and CRP (0.83). The ROC analysis showed that at a cut-off value 647 pg/ml, presepsin may be able to discriminate between patients with and without sepsis with a sensitivity and a specificity of 92.9% and 83.3% respectively with 95% confidence intervals of 0.929 (0.805 to 0.985). And the presepsin values were significantly higher in the patients with the more severe septic condition (for example, sepsis, severe sepsis, septic shock). In addition, a significant correlation was found between the SOFA scores and the presepsin values (*r*^2 ^= 0.258; *P *< 0.01). But there was only weak correlation between APACHE II scores and the presepsin values (*r*^2 ^= 0.053).

## Conclusion

In this study, presepsin is the most valuable predictor about sepsis compared with PCT, IL-6, and CRP. Moreover, these results suggest that presepsin values can serve as a parameter that closely reflects the pathology. So we strongly suggest that presepsin will be not only a very useful new biomarker for a diagnosis of the sepsis, but also useful for monitoring the severity of the disease in the near future.
